# Mechanisms of Fasting-Mediated Protection against Renal Injury and Fibrosis Development after Ischemic Acute Kidney Injury

**DOI:** 10.3390/biom9090404

**Published:** 2019-08-22

**Authors:** Pedro Rojas-Morales, Edilia Tapia, Juan Carlos León-Contreras, Susana González-Reyes, Angélica Saraí Jiménez-Osorio, Joyce Trujillo, Natalia Pavón, Jessica Granados-Pineda, Rogelio Hernández-Pando, Laura Gabriela Sánchez-Lozada, Horacio Osorio-Alonso, José Pedraza-Chaverri

**Affiliations:** 1 Departamento de Biología, Facultad de Química, Universidad Nacional Autónoma de México, Ciudad de México 04510, Mexico; 2 Departamento de Fisiopatología Cardio-Renal, Instituto Nacional de Cardiología Ignacio Chávez, Ciudad de México 14080, Mexico; 3 Departamento de Patología, Instituto Nacional de Ciencias Médicas y Nutrición Salvador Zubirán, Ciudad de México 14080, Mexico; 4 Laboratorio de Bioquímica, Facultad de Medicina y Psicología, Universidad Nacional Autónoma de Baja California, Tijuana 22390, Mexico; 5 Laboratorio de Medicina Genómica, Hospital Regional Lic. Adolfo López Mateos, ISSSTE, Ciudad de México 01030, Mexico; 6 Consejo Nacional de Ciencia y Tecnología-Consorcio de Investigación, Innovación y Desarrollo para las Zonas Áridas- Instituto Potosino de Investigación Científica y Tecnológica, San Luis Potosí 78216, Mexico; 7 Departamento de Farmacología, Instituto Nacional de Cardiología Ignacio Chávez, Ciudad de México 14080, Mexico

**Keywords:** fasting, fibrosis, mitochondrial dysfunction, oxidative stress, inflammation, ER stress, ischemia-reperfusion injury

## Abstract

Ischemia-reperfusion injury of the kidney may lead to renal fibrosis through a combination of several mechanisms. We recently demonstrated that fasting protects the rat kidney against oxidative stress and mitochondrial dysfunction in early acute kidney injury, and also against fibrosis development. Here we show that preoperative fasting preserves redox status and mitochondrial homeostasis at the chronic phase of damage after severe ischemia. Also, the protective effect of fasting coincides with the suppression of inflammation and endoplasmic reticulum stress, as well as the down-regulation of the mechanistic target of rapamycin (mTOR) and extracellular signal-regulated kinase 1/2 (ERK1/2) signaling pathways in the fibrotic kidney. Our results demonstrate that fasting targets multiple pathophysiological mechanisms to prevent renal fibrosis and damage that results after renal ischemia-reperfusion injury.

## 1. Introduction

Fibrosis is a hallmark and a driver of chronic kidney disease (CKD) [1]. It is a maladaptive repair process after severe acute kidney injury (AKI), leading to end-stage renal disease and increased mortality [2,3,4]. The molecular signature predisposing to fibrosis development after AKI is not fully understood. Multiple interrelated factors such as oxidative stress, mitochondrial dysfunction, endoplasmic reticulum (ER) stress, and an exacerbated inflammatory response contribute to extracellular matrix deposition, loss of kidney architecture, disruption of cellular homeostasis, and finally renal dysfunction [4,5,6,7,8,9]. Mechanistically, transforming growth factor β (TGF-β1) has been shown to play a direct role in fibrogenesis, as inhibition of TGF-β1 signaling by genetic or pharmacological means significantly abrogates fibrosis progression after severe kidney injury [10]. Emerging evidence also implicates extracellular signal-regulated kinase 1/2 (ERK1/2) and the mechanistic target of rapamycin (mTOR), which operate downstream of TGF-β1 signaling, in renal fibrosis development [11,12], thus expanding our understanding of CKD pathophysiology.

Mild food restriction has been known for many years to lead to multisystemic beneficial effects and activates cellular stress response pathways to protect cells and tissues from damage [13,14]. As such, dietary restriction regimens, namely calorie restriction, fasting, and intermittent fasting, confer protection against ischemic insults in organs like the liver, heart, brain, and the kidney [14,15]. By using a rat model of AKI induced by ischemia-reperfusion (IR), we recently demonstrated that preoperative fasting protects the kidney against redox and mitochondrial alterations seen early after IR, and also confers long-lasting protection against fibrosis development [16]. These findings led us to investigate the mechanisms that preoperative fasting regulates to protect against the chronic phase of damage induced by ischemia. To this end, we first tested the efficacy of fasting against fibrogenesis in a slightly different, yet more severe IR injury model than in our previous work. Then, oxidative stress, mitochondrial homeostasis, ER stress, inflammation, as well as the signal transducers mTOR and ERK1/2 were assessed two weeks later to understand the magnitude of the protective effect of fasting.

## 2. Materials and Methods 

### 2.1. Reagents, Materials, and Antibodies

All chemicals used in this study were purchased from Sigma-Aldrich (St Louis, MO, USA). Commercial kits to measure blood urea nitrogen (BUN), creatinine, and glucose levels in plasma were obtained from SpinReact (Girona, Spain). The nitrocellulose membrane, the Chemiluminiscent Detection Kit, and the primary antibody for peroxisome proliferator-activated receptor gamma coactivator 1 alpha (PGC1α, #AB3242, 1:2,000) were from Merck Millipore (Burlington, MA, USA). The antibody against catalase (CAT, #219010, 1:2,500) was purchased from Calbiochem (San Diego, CA, USA). Antibodies against activating transcription factor 4 (ATF4, #GTX30070, 1:5,000), alpha smooth muscle actin (α-SMA, #GTX100034, 1:2,000), glutamate-cysteine ligase modifier subunit (GCLM, #GTX114075, 1:2,500), and glyceraldehyde-3-phosphate dehydrogenase (GAPDH, #GTX100118, 1:10,000) were purchased from GeneTex (Irvine, CA, USA). The antibody against nuclear factor kappa B p65 (NF-κB p65, #PA5-16545, 1:2,000) was purchased from Thermo Fisher Scientific (Waltham, MA, USA). The antibodies against NADH:Ubiquinone oxidoreductase subunit B8 (NDUFB8, #ab110242, 1:5,000), succinate dehydrogenase subunit B (SDHB, #ab14714, 1:5,000), cytochrome c oxidase subunit 1 (MTCO1, #ab14705, 1:2,000), ATP synthase F1 subunit α (ATP5A, #ab14748, 1:10,000), 8-hydroxy-2′-deoxyguanosine (8-OHdG, #ab62623, 1:200), and nucleotide-binding oligomerization domain (NOD)-like receptor protein 3 (NLRP3, #ab214185, 1:1,000) were from Abcam (Cambridge, MA, USA). Antibodies against mitofusin 2 (NFN2 #9482S, 1:2,000), phospho-ERK1/2 (p-ERK1/2, #4370, 1:2,000), ERK1/2 (#9102, 1:2,000), and phospho-S6 kinase (p-S6K, #9205, 1:2,000) were from Cell Signaling (Danvers, MA, USA). Antibodies against dynamin-related protein 1 (DRP1, #sc-32898, 1:1,000), interleukin 6 (IL-6, #sc-57315, 1:500), TGF-β1 (#sc-130348, 1:1,000), tumor necrosis factor alpha (TNFα, #sc-52746, 1:100) were from Santa Cruz Biotechnology (Dallas, TX, USA). Antibodies against collagen IV (Col IV, #SAB5200500, 1:4,000), glucose-regulated protein 94 (GRP94, #G4420, 1:2,000), and protein kinase RNA-like endoplasmic reticulum kinase (PERK, #P0074, 1:2,000) were from Sigma-Aldrich. Antibody against nicotinamide adenine dinucleotide phosphate oxidase 4 (NOX4, #NB110-58849, 1:1,000) was from Novus Biologicals (Centennial, CO, USA). Anti-rabbit (#7074S, 1:5,000) and anti-mouse (#7076S, 1:5,000) horseradish peroxidase (HRP)-conjugated secondary antibodies were purchased from Cell Signaling (Danvers). Goat polyclonal secondary antibody to mouse immunoglobulin G (Alexa Fluor 647, #ab150115, 1:1,000) was purchased from Abcam. 

### 2.2. Animals, Experimental Design, and Study Approval

**Figure 1:**
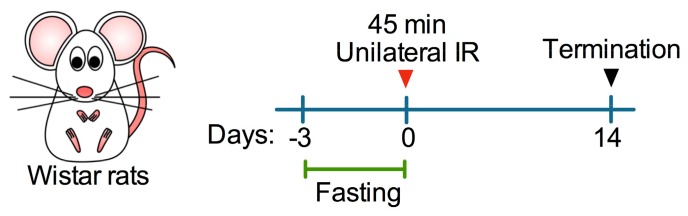
Schematic depiction of the experimental schedule. Fed or fasted rats were subjected to either sham surgery or 45 min of unilateral ischemia-reperfusion (IR). After surgery, all rats were fed and euthanized 14 days after IR.

Eight nine-week-old male Wistar rats weighing 240–260 g were used in this study. Rats were maintained in standard laboratory conditions with normal rodent chow diet and water provided ad libitum until the beginning of experiments. After acclimatization for at least one week, rats were randomly assigned to either one of the following experimental groups (*n* = 4, each): sham control, IR only, and IR + Fasting. Rats enrolled in the IR + Fasting group were deprived of food (beginning at 10:00 am) for three days before surgery. Rats were subjected to unilateral renal ischemia for 45 min and then allowed to recover for two weeks (Figure 1). All animal experiments were approved by the Institutional Animal Care and Use Committee of Instituto Nacional de Cardiología Ignacio Chávez (#INC/CICUAL/005/2018).

### 2.3. Unilateral Ischemia-Reperfusion Injury 

A unilateral IR injury model was used to induce severe tubular damage and fibrosis while avoiding unacceptable high mortality seen in bilateral IR injury models [17]. Fed or fasted rats were anesthetized with an intraperitoneal injection of sodium pentobarbital (60 mg/kg), and after exposing the left kidney through a midline abdominal incision, the left renal artery was occluded for 45 min using nontraumatic vascular clamps. Laparotomy without ischemia was performed in sham-operated rats, which served as control. After ischemia or sham operation, all rats were fed ad libitum and euthanized two weeks later to harvest blood and kidneys for histology, transmission electron microscopy, immunohistochemistry, and Western Blot analysis.

### 2.4. Plasma Biochemistry 

The levels of blood urea nitrogen (BUN), creatinine, and glucose were measured in plasma samples using commercial kits according to the manufacturer’s instructions. 

### 2.5. Histology and Transmission Electron Microscopy

To assess kidney damage and fibrosis, kidneys were immersed in a 4% paraformaldehyde plus 1.5% glutaraldehyde solution, embedded in paraffin, sectioned at 4 µm, and stained with routine hematoxylin and eosin (H&E) or Masson trichrome. For automated morphometry, six random choice fields for each kidney were studied at 200× magnification (total area 3,1 × 10^5^ μm^2^), the total number of convoluted tubules and the percentage of atrophic or cystic tubules was determined using ImageJ (National Institute of Health, Bethesda, MD, USA), and the data were expressed as the percentage of atrophic tubules. For fibrosis determination, the same software program and the Masson trichrome stain were used, the total area at 200× magnification was measured in six random choice fields for each section and the percentage of interstitial fibrosis was determined from each rat.

For mitochondrial morphology visualization, paraformaldehyde/glutaraldehyde-fixed kidney fragments were post-fixed with 2% osmium tetroxide, embedded in epoxy resin, ultra-sectioned at 70–90 nm, and stained with uranyl acetate and lead citrate. Images were acquired with the Tecnai Spirit BioTWIN transmission electron microscope (Hillsboro, OR, USA).

### 2.6. Immunohistochemistry and Immunofluorescence

For immunodetection of DNA damage, paraffin-embedded kidney sections were dewaxed, rehydrated, and incubated for 2 h with the anti-8-OHdG (1:200) antibody. After several washes, tissue sections were incubated with an HRP-conjugated secondary antibody, revealed with 3,3′-diaminobenzidine and counterstained with hematoxylin. For immunofluorescence, kidney sections were incubated for 2 h with a primary antibody against TNFα (1:100) and then with the Alexa Fluor 647. Nuclei were stained with 1 µg/mL 4′,6-diamidino-2-fenilindol (DAPI). Images were acquired with an epifluorescence microscope. 

### 2.7. Subcellular Fractionation 

Mitochondria were isolated by differential centrifugation essentially as previously described [16]. Frozen kidney fragments were homogenized in ice-cold buffer containing 225 mM d-mannitol, 75 mM sucrose, 10 mM 4-(2-hydroxyethyl)-1-piperazineethanesulfonic acid (HEPES), 1 mM ethylenediamine tetraacetic acid (EDTA), and 0.5 mg/mL fatty acid-free bovine serum albumin (BSA), pH 7.4, using a Potter–Elvehjem tissue homogenizer, and then centrifuged at 800× *g* for 10 min. The obtained supernatant was separated into a new tube and centrifuged again at 10,000× *g* for another 10 min to obtain mitochondria (pellet) and cytosol (supernatant), which were prepared for western blot analysis.

### 2.8. Western Blot

Kidneys were homogenized in ice-cold lysis buffer containing 50 mM Tris pH 8.0, 150 mM NaCl, 5 m EDTA, 1 mM ethylene glycol-bis(2-aminoethylether)-N N N′N-tetraacetic acid (EGTA), sodium deoxycholate, 0.1% sodium dodecyl sulfate (SDS), 1% Nonidet P-40, and protease and phosphatase inhibitors, and then centrifuged at 15,000× *g* for 10 min at 4 °C. Twenty µg of total protein were subjected to polyacrylamide gel electrophoresis and subsequently transferred at 100 volts for 1.5 h to nitrocellulose membranes, which were then incubated for 2 h at room temperature with primary antibodies. After several washes, membranes were incubated at room temperature for 1 h with secondary antibodies, and specific proteins were detected by chemiluminescence, quantified using ImageJ (National Institute of Health, Bethesda, MD, USA), normalized to GAPDH, and finally expressed as fold-change vs. sham. For protein normalization, membranes were incubated at room temperature for 30 min with a stripping solution (100 mM glycine pH 2.5, 0.5% SDS), and then re-probed with anti-GAPDH.

### 2.9. Enzyme-Linked Immunosorbent Assays

Kidney homogenates were obtained, and the levels of interleukin 1 beta (IL-1β), IL-6, and TNFα were measured with an enzyme-linked immunosorbent assay (ELISA) (PeproTech, Rocky Hill, NJ, USA). 

### 2.10. Statistical Analysis

Data are presented as mean ± standard error of the mean (SEM). Differences between groups were assessed by one-way analysis of variance (ANOVA) followed by the Tukey test using the GraphPad Prism 6 software (San Diego, CA, USA). The level of significance was set at *p* < 0.05.

## 3. Results

### 3.1. Fasting Attenuates Tubular Injury and Fibrosis Induced by Ischemia-Reperfusion Injury

**Figure 2:**
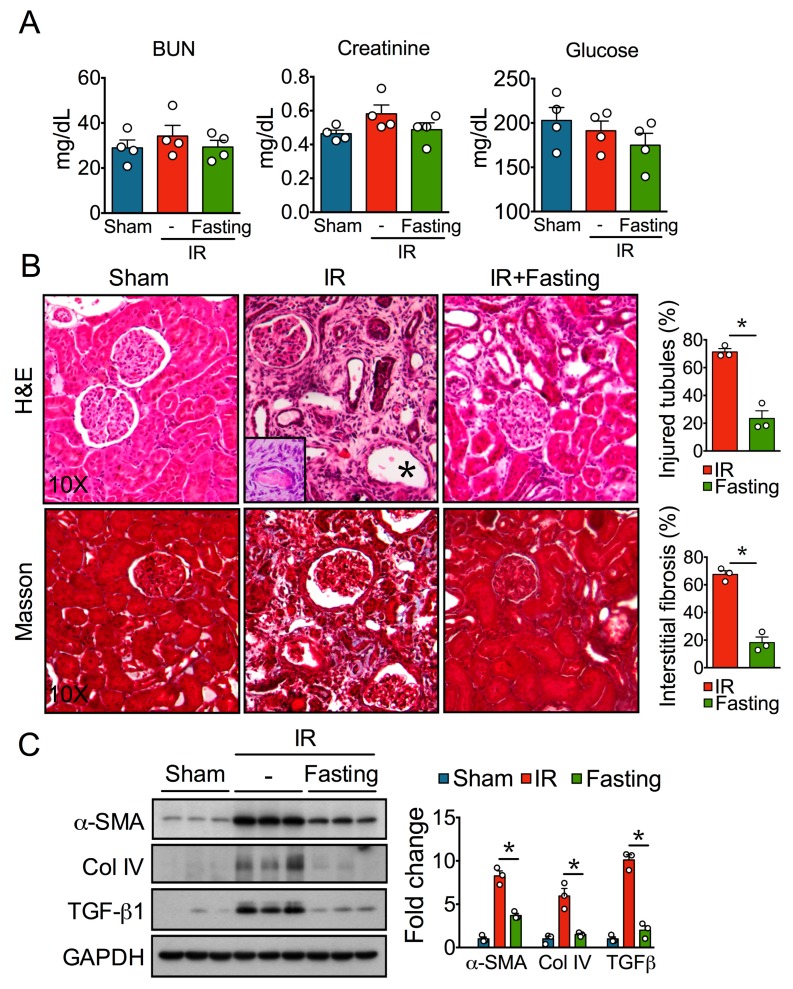
Effect of fasting on renal injury and development of fibrosis after two weeks of unilateral ischemia-reperfusion (IR) injury. (**A**) Plasma levels of blood urea nitrogen (BUN), creatinine, and glucose assessed 14 days post-IR or sham operation. (**B**) Representative hematoxylin & eosin (H&E)- and Masson trichrome-stained kidney sections (left) at 10× magnification from sham, IR, and IR + Fasting experimental groups at day 14 post-IR. Quantification (right) of injured tubules (H&E) and interstitial fibrosis (Masson) in IR and IR + Fasting experimental groups. In contrast to the normal kidney, histology showed by a rat from the sham group, kidney sections from the IR animal show extensive interstitial fibrosis with mild chronic inflammatory infiltrate, and numerous atrophic small tubules coated with cuboidal epithelial cells or with cystic appearance (asterisk). The inset shows a high-power micrograph of the interstitial fibrotic process, with numerous fusiform cells that correspond to fibroblasts around an atrophic tubule with the hyaline cast. Fasting decreased interstitial fibrosis and tubular atrophy. (**C**) Immunoblots (left) and quantification (right) of alpha-smooth muscle actin (α-SMA), collagen IV (Col IV), and transforming growth factor β1 (TGF-β1) in the kidney cortex of rats from sham, IR, and IR + Fasting experimental groups. GAPDH, glyceraldehyde-3-phosphate dehydrogenase. Data are expressed as mean ± standard error of the mean (SEM). *n* = 3 to 4 animals per group. * *p* < 0.05.

Severe ischemic AKI results in chronic renal injury and interstitial fibrosis [2]. Rats studied two weeks after 45 min of unilateral renal ischemia presented no changes in BUN, creatinine, and plasma glucose (Figure 2A); however, they showed severe renal damage demonstrated by histological analysis of kidney sections H&E stained (Figure 2B). Kidney sections from the sham group exhibited normal morphology. In contrast, post-IR kidneys showed extensive tubular atrophy, manifested by cuboidal or flattened epithelium that was part of small or cystic tubules, many other tubules showed detachment of epithelial cells or hyaline casts; some glomeruli showed retracted capillary tuffs and were surrounded by fibrotic interstitium with chronic inflammatory infiltrate and numerous fibroblasts (Figure 2B). Masson Trichrome staining confirmed extensive tubule-interstitial fibrosis (Figure 2B), and automated morphometry showed significantly greater tubular damage and interstitial fibrosis in the kidneys of IR group. In addition, Western blot analysis showed increased protein expression of fibrosis markers such as TGF-β1, α-SMA, and Col IV (Figure 2C). In contrast, such increased damage and fibrotic index after IR were not observed in the kidneys of rats that were preoperatively fasted for three days (Figure 2B,C), indicating that fasting confers strong protection against tubular injury and fibrosis, which is consistent with our previous report [16].

### 3.2. Fasting Prevents Oxidative Stress Long after Ischemia-Reperfusion Injury

**Figure 3:**
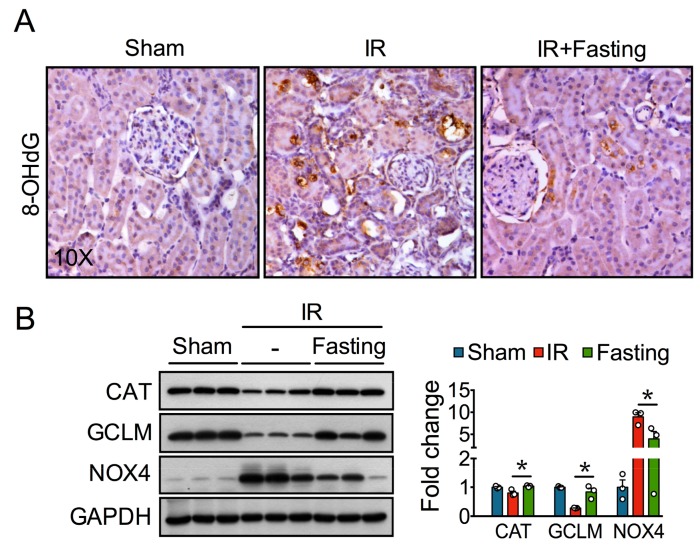
Fasting protects against oxidative stress resulting after two weeks of unilateral ischemia-reperfusion (IR) injury. (**A**) Representative kidney sections (10×) immunostained for 8-hydroxy-2′-deoxyguanosine (8-OHdG) in sham, IR, and IR + Fasting experimental groups at day 14 post-IR or sham surgery. (**B**) Immunoblots (left) and quantification (right) of catalase (CAT), glutamate-cysteine ligase modifier subunit (GCLM), and nicotinamide adenine dinucleotide phosphate oxidase 4 (NOX4) in the kidney cortex of rats from sham, IR, and IR + Fasting experimental groups. GAPDH, glyceraldehyde-3-phosphate dehydrogenase. Data are expressed as mean ± SEM. *n* = 3 animals per group. * *p* < 0.05.

Our previous study showed fasting protects against redox imbalance seen early (24 h) after ischemic AKI [16]. Increased oxidative deoxyribonucleic acid (DNA) damage, revealed by 8-OHdG immunostaining, was observed two weeks post-IR (Figure 3A), suggesting ischemia leads to persistent oxidative stress. Preoperative fasting reduced the oxidative damage induced by IR injury (Figure 3A), which was associated with increased protein levels of the hydrogen peroxide (H_2_O_2_)-degrading enzyme CAT and GCLM, the regulatory subunit of the glutamate-cysteine ligase enzyme involved in glutathione synthesis (Figure 3B). On the other hand, fasting reduced NOX4 protein expression, a major renal source of reactive oxygen species (ROS) [18] (Figure 3B). Thus, these data indicate fasting conferred long-lasting protection against IR-induced oxidative stress by activating antioxidant defenses and by down-regulating NOX4.

### 3.3. Fasting Protects Mitochondria Long After Ischemia-Reperfusion Injury

**Figure 4:**
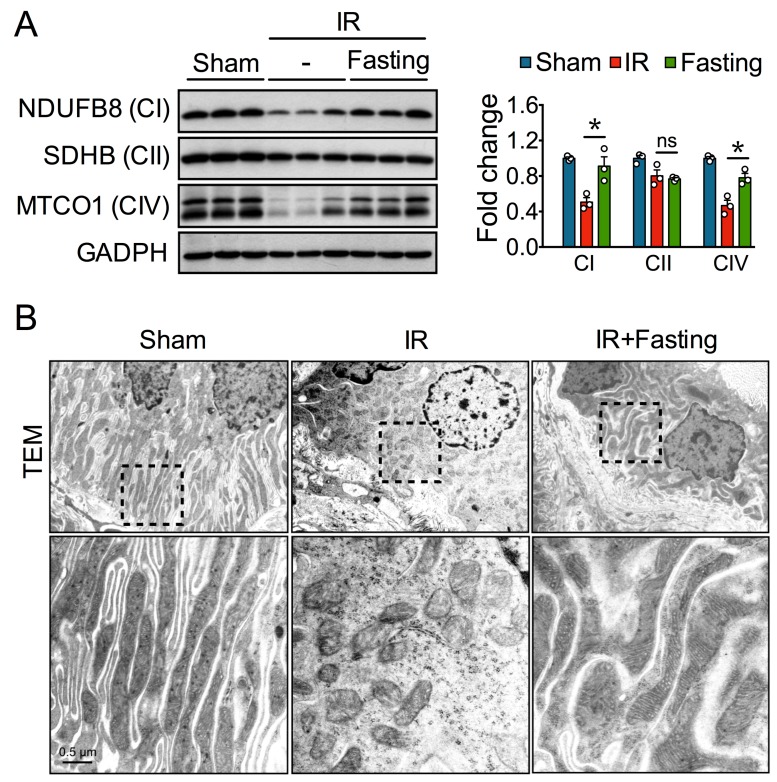
Fasting protects mitochondria long after ischemia-reperfusion (IR) injury. (**A**) Protein expression (left) and quantification (right) of respiratory-complex subunits in kidneys from fed or fasted rats two weeks after IR injury. NDUFB8, NADH:Ubiquinone oxidoreductase subunit B8; SDHB, succinate dehydrogenase subunit B; MTCO1, cytochrome c oxidase subunit 1; GAPDH, glyceraldehyde-3-phosphate dehydrogenase; ns, not significant. (**B**) Representative transmission electron microscopy (TEM) micrographs of mitochondria from proximal tubular epithelial cells two weeks after IR. Magnified areas (Insets) are taken from the black boxes with dash lines. Data are expressed as mean ± SEM. *n* = 3 animals per group. * *p* < 0.05.

Altered mitochondrial homeostasis has been observed long after severe ischemia [6]. Whereas IR reduced the levels of NDUFB8, SDHB, and MTCO1 oxidative phosphorylation (OXPHOS) proteins, fasting increased the levels of NDUFB8 and MTCO1, although not that of SDHB (Figure 4A). Consistent with these favorable changes, transmission electron microscopy showed that cristae effacement and fragmented mitochondria in proximal tubular epithelial cells from post-IR kidneys were also reduced by fasting (Figure 4B).

**Figure 5:**
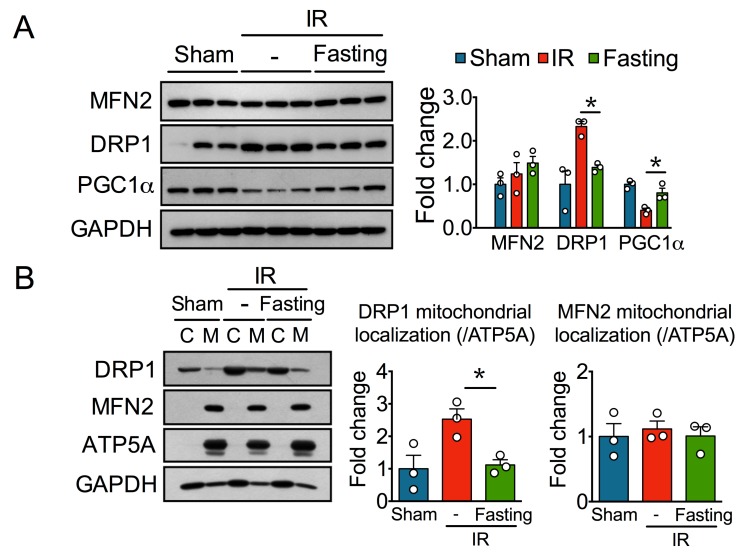
Fasting regulates disturbances in mitochondria dynamics and biogenesis induced by ischemia-reperfusion (IR) injury. (**A**) Immunoblots (left) and quantification (right) of mitofusin 2 (MFN2), dynamin-related protein 1 (DRP1), and peroxisome proliferator-activated receptor gamma coactivator 1 alpha (PGC1α) in total kidney homogenates of rats from sham, IR, and IR + Fasting experimental groups. (**B**) Representative immunoblots (left) and quantification (right) of DRP1 and MFN2 in isolated mitochondria from the experimental groups described. (**C**) cytoplasmic fraction; M, mitochondrial fraction; ATP5A, ATP synthase F1 subunit alpha; GAPDH, glyceraldehyde-3-phosphate dehydrogenase. Data are expressed as mean ± SEM. *n* = 3 animals per group. * *p* < 0.05.

Mitochondrial biogenesis and dynamics are required for maintaining a healthy mitochondrial pool and hence mitochondrial homeostasis [19]; therefore, we further assessed the levels of the mitochondrial fusion protein mitofusin 2 (MFN2), the mitochondrial fission protein dynamin-related protein 1 (DRP1), and PGC1α, a master transcriptional regulator of mitochondrial biogenesis [19]. While MFN2 remained mostly unaltered, DRP1 significantly increased in the IR group, and this increase was diminished by fasting. Also, we observed reduced levels of PGC1α in the IR group, but not in the fasted group (Figure 5A). To confirm the involvement of DRP1 in mitochondrial fragmentation after IR injury, we performed cellular fractionation and analyzed mitochondrial localization of DRP1. IR increased the levels of DRP1 at the mitochondria fraction, and this effect was prevented by fasting (Figure 5B). Again, we did not observe changes in MFN2. Thus, our results suggest that fasting partially protects mitochondria in renal fibrosis by regulating mitochondrial biogenesis and dynamics.

### 3.4. Fasting Protects Against Ischemia-Reperfusion Induced Endoplasmatic Reticulum Stress

**Figure 6:**
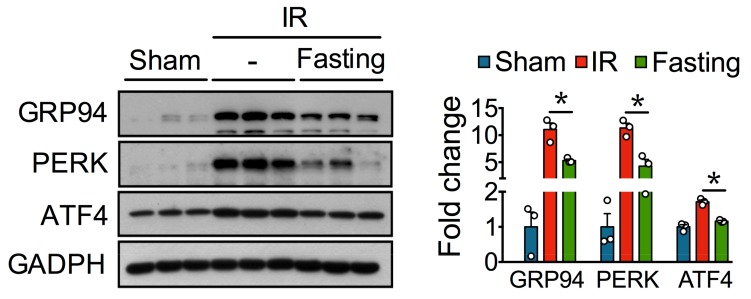
Fasting reduces endoplasmic reticulum (ER) stress induced by ischemia-reperfusion (IR) injury. Immunoblots (left) and quantification (right) of glucose-regulated protein 94 (GRP94), protein kinase RNA-like endoplasmic reticulum kinase (PERK), and activating transcription factor 4 (ATF4) in the kidney from the experimental groups described. GAPDH, glyceraldehyde-3-phosphate dehydrogenase. Data are expressed as mean ± SEM. *n* = 3 animals per group. * *p* < 0.05.

Emerging evidence indicates ER stress may contribute to fibrosis [7]. Perturbed ER homeostasis was observed in fibrotic kidneys. Unfolded protein response markers, such as GRP94, PERK, and ATF4, were increased in the IR group, compared to the non-fibrotic kidneys from the control sham-operated rats (Figure 6). Fasting, on the other hand, diminished ER stress proteins, thus suggesting that fasting maintains protein folding and quality control in post-ischemic kidneys.

### 3.5. Fasting Suppresses Inflammation Long After Ischemia-Reperfusion Injury

**Figure 7:**
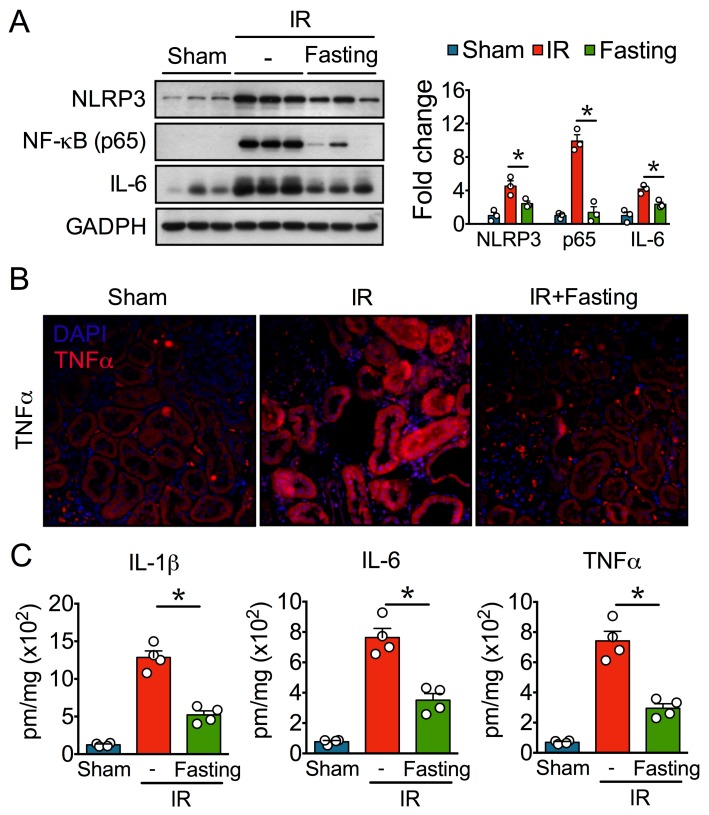
Fasting suppresses persistent inflammation induced by ischemia-reperfusion (IR) injury. (**A**) Immunoblots (left) and quantification (right) of nucleotide-binding oligomerization domain (NOD)-like receptor protein 3 (NLRP3) inflammasome, nuclear factor kappa B (NF-κB), and interleukin 6 (IL-6) in fed or fasted rats subjected to ischemia-reperfusion (IR). GAPDH, glyceraldehyde-3-phosphate dehydrogenase. (**B**) Representative kidney sections immunostained for tumor necrosis factor alpha (TNFα) and analyzed by fluorescence microscopy. (**C**) Renal content of interleukin 1 beta (IL-1β), IL-6, and TNFα. DAPI, 4′,6-diamidino-2-fenilindol. Data are expressed as mean ± SEM. *n* = 3 to 4 animals per group. * *p* < 0.05.

Chronic inflammation is tightly associated with fibrosis development [20], so we evaluated the effect of IR and fasting on inflammatory markers. Our Western blot analysis showed that IR increased protein levels of the NLRP3 inflammasome, NF-κB, and IL-6 (Figure 7A), indicative of significant inflammation. Immunofluorescence microscopy revealed high levels of TNFα in renal tubular epithelial cells after IR injury (Figure 7B). In addition, IR increased the renal content of IL-1β, IL-6, and TNFα, revealed by ELISA assays (Figure 7C). On the other hand, preoperative fasting for three days diminished inflammatory markers, thus indicating that fasting exerts strong protection against chronic inflammation after IR.

### 3.6. Effect of Fasting on ERK and mTOR Signaling Pathway

**Figure 8:**
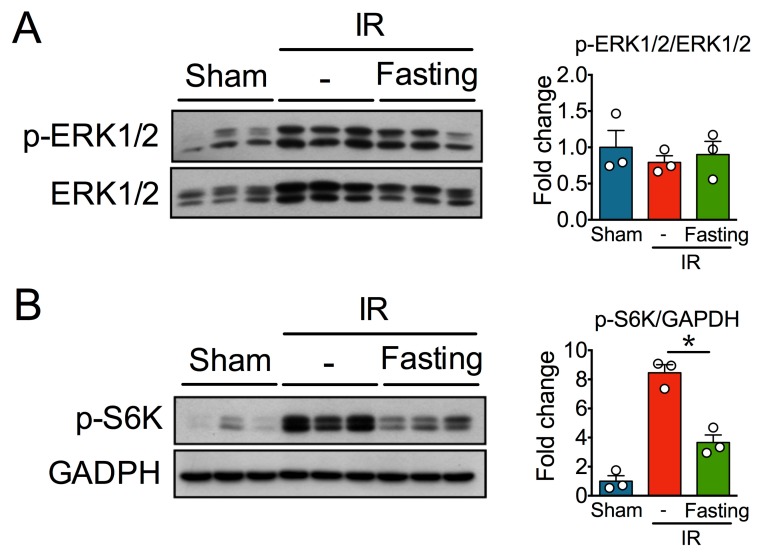
Effect of fasting on ischemia-reperfusion (IR)-associated signaling pathways. (**A**) Immunoblots (left) and quantification (right) of phospho-extracellular-regulated kinase 1/2 (p-ERK1/2) and ERK1/2 in the kidney cortex of rats from sham, IR, and IR + Fasting experimental groups. (**B**) Immunoblots (left) and quantification (right) of phospho-S6 kinase (p-S6K). GAPDH, glyceraldehyde-3-phosphate dehydrogenase. Data are expressed as mean ± SEM. *n* = 3 animals per group. * *p* < 0.05.

Several signaling pathways have been implicated in fibrosis development after ischemia, and various studies clearly indicate that both ERK1/2 and mTOR have multiple crosstalks with the pathophysiological mechanisms operating in CKD [11,12]. Although we observed that IR increased the levels of both phosphorylated and non-phosphorylated ERK1/2, densitometry analysis of the p-ERK1/2 to ERK1/2 ratio revealed no activation of these kinases after IR (Figure 8A). Also, although fasting diminished the levels of p-ERK1/2, it did not modify p-ERK1/2 to ERK1/2 ratio. On the other hand, we observed that the levels of the phosphorylated form of S6K, a downstream target and surrogated indicator of active mTOR, were significantly upregulated in fibrotic kidneys (Figure 8B). Fasting, on the other hand, diminished the levels of phospho-S6K. 

## 4. Discussion

It is clear that renal injury and development of fibrosis are mediated by an interplay of several mechanisms. For example, TGF-β1, a key regulator of extracellular matrix assembly and remodeling, promotes activation of NOX4 and down-regulation of antioxidant defenses, leading to oxidative stress and damage [21,22]. Redox imbalances, in turn, leads to mitochondrial dysfunction, and in turn, damaged mitochondria produce excessive free radicals [23]. Also, oxidative stress perturbs ER homeostasis and leads to proteotoxic stress, affecting again mitochondrial functioning and ultimately amplifying ROS generation [24]. In addition, sustained oxidative stress, mitochondrial dysfunction, and ER stress can independently contribute to chronic inflammation and activate fibrogenic signals [4,5,22,25,26], thus creating a vicious cycle that culminates in kidney malfunctioning and death. 

Dietary restriction has been widely recognized as a strong non-genetic, non-pharmacological tool to increase multisystemic protection against stress and to postpone many aspects related to the onset, rate, and progression of age-related chronic diseases, mainly due to its antioxidant and anti-inflammatory properties [27]. However, we know very little about how dietary restriction protocols impact kidney physiology and disease. We and others have shown that fasting is renoprotective in early AKI, as this short-term dietary intervention diminishes tubular injury, cell death, inflammation, oxidative stress, and mitochondrial dysfunction seen 24 h after IR [16,28]. In the present work, we focused on the effect of preoperative fasting on tubular injury and renal fibrosis that develop long after AKI.

We used a unilateral renal IR without contralateral nephrectomy injury model that leads to tubular injury and interstitial fibrosis, to analyze the long-term protective effect of fasting. As expected, because in this model, rodents still have one intact kidney, no changes in plasma urea and creatinine were observed. However, at two weeks after 45 min of unilateral IR, fibrosis, oxidative stress, mitochondrial dysfunction, ER stress, and inflammation were prominent, and histology revealed severe tubular injury. Also, renal injury was accompanied by increased levels of phospho-ERK1/2 and phospho-S6K, which are engaged in fibrogenic processes. A recent study reported that pharmacological inhibition of ERK1/2 signaling significantly ameliorates collagen deposition and myofibroblast expansion during kidney fibrosis, a phenomenon that is explained by reduced mTOR, signal transducer and activator of transcription 3 (STAT3), small mothers against decapentaplegic 2/3 (Smad2/3), and NF-κB pathway activation [11]. 

**Figure 9:**
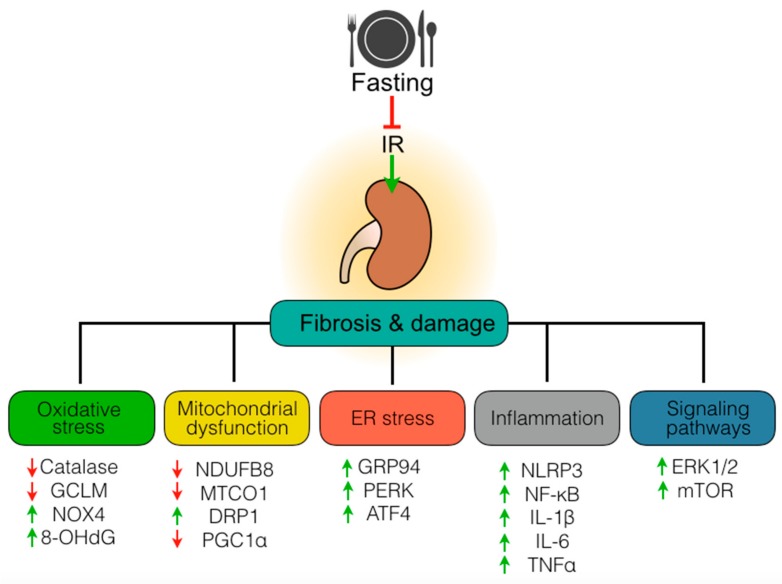
Protection against chronic damage and fibrosis development after ischemia-reperfusion (IR) injury by fasting. Preoperative fasting limits IR-induced fibrosis and renal damage by suppressing oxidative stress, mitochondrial dysfunction, endoplasmic reticulum (ER) stress, inflammation, and signaling pathways. GCLM, glutamate-cysteine ligase modifier subunit; NOX4, nicotinamide adenine dinucleotide phosphate oxidase 4; 8-OHdG, 8-hydroxy-2′-deoxyguanosine; NDUFB8, NADH:Ubiquinone oxidoreductase subunit B8; MTCO1, cytochrome c oxidase subunit 1; succinate dehydrogenase subunit B; DRP1, dynamin-related protein 1; PGC1α, peroxisome proliferator-activated receptor gamma coactivator 1 alpha; GRP94, glucose-regulated protein 94; PERK, protein kinase RNA-like endoplasmic reticulum kinase; ATF4, activating transcription factor 4; NLRP3, NOD-like receptor protein 3; NF-κB, nuclear factor kappa B; IL-1β, interleukin 1 beta; IL-6, interleukin 6; TNFα, tumor necrosis factor-alpha; ERK1/2, extracellular signal-regulated kinase 1/2; mTOR, mechanistic target of rapamycin.

We found that fasting primes the kidney to resist tubular injury and fibrosis development through various mechanisms (Figure 9). These include preservation of redox balance, maintenance of both mitochondria and ER homeostasis and suppression of inflammatory processes persisting long after the initial ischemic insult. Also, fasting abrogated ERK1/2 and mTOR signaling, both playing important roles in renal fibrosis development. Overall, our data provide further evidence into the long-term benefit of fasting preconditioning. Interestingly, others have shown that food restriction protects against polycystic kidney disease, a leading genetic cause of end-stage renal disease [29]. Also, protein restriction protects against renal IR injury [30] and ameliorates CKD in uremic rats [31]. Together, these findings illustrate how dietary approaches, other than pharmacological ones, may prove useful in improving the outcome of kidney diseases [32]. 

## 5. Conclusions

Fasting is a strong nutritional intervention to abrogate multiple pathophysiological mechanisms implicated in tissue injury and fibrosis that develops after renal IR.
